# Supplementary material for: CHESS 3: an improved, comprehensive catalog of human genes and transcripts based on large-scale expression data, phylogenetic analysis, and protein structure
Source: Genome Biol. 2023 Oct 30;24:249. doi: 10.1186/s13059-023-03088-4 (PMC10614308; doi:10.1186/s13059-023-03088-4)
Supplement: Supplementary file 1 — Additional file 1: Figure S1. Change in the alignment rate of the GTEx samples between the alignment strategy implemented when building the earlier CHESS2 database (blue), and the improved alignment protocol using an updated HISAT2 release, sex-specific genome indices, and newer guide annotation (red). Figure S2. Number of RefSeq XM and NM transcripts present in different datasets, including CHESS 3, GENCODE, Assembled (which includes all transcripts assembled from GTEx data), and TieBrush (which includes all transcripts retained after filtering the Assembled set with TieBrush). All of the transcripts were contained in RefSeq, which is shown in blue for comparison. Figure S3. Upset plot showing overlap of transcripts in six different datasets: CHESS, RefSeq, GENCODE, MANE, all transcripts assembled from GTEx (“Assembled”), and TieBrush-filtered GTEx isoforms. The plot illustrates the number of transcripts unique to each dataset, as well as the number shared between different datasets. Combinations with no transcripts are excluded from the figure as well as transcripts that were unique to raw assemblies of GTEx and TieBrush. The 136 transcripts that were assembled and present in both RefSeq and GENCODE, but not in CHESS, were removed because they either lacked a valid ORF or because the translated protein was too short compared to other isoforms, as explained in the main text. [file 13059_2023_3088_MOESM1_ESM.docx]

**Supplementary Figures**


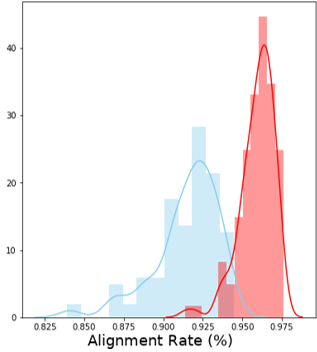


**Figure S1.** Change in the alignment rate of the GTEx samples between the alignment strategy implemented when building the earlier CHESS2 database (blue), and the improved alignment protocol using an updated HISAT2 release, sex-specific genome indices, and newer guide annotation (red).

**
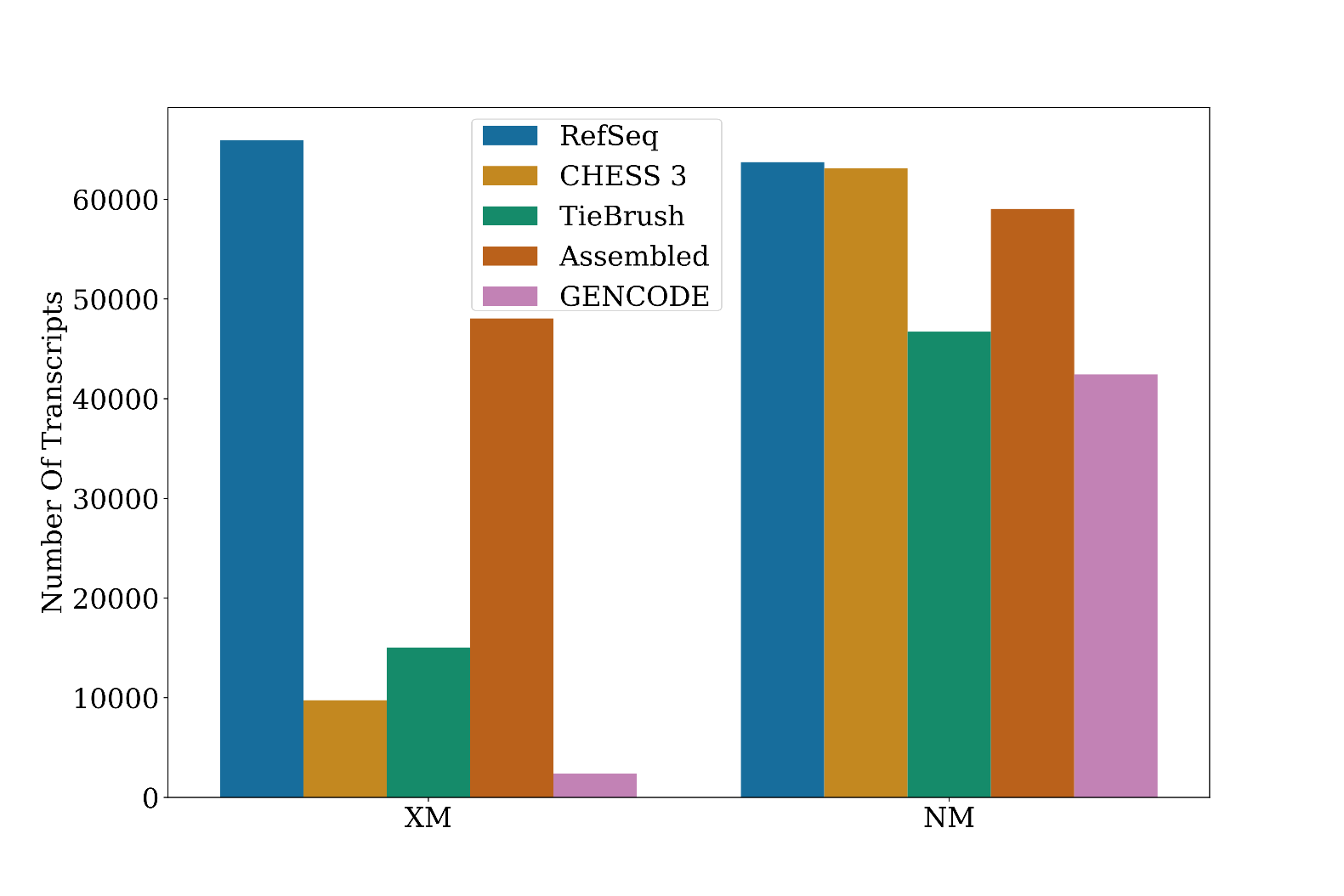
Figure S2.** Number of RefSeq XM and NM transcripts present in different datasets, including CHESS 3, GENCODE, Assembled (which includes all transcripts assembled from GTEx data), and TieBrush (which includes all transcripts retained after filtering the Assembled set with TieBrush). All of the transcripts were contained in RefSeq, which is shown in blue for comparison.


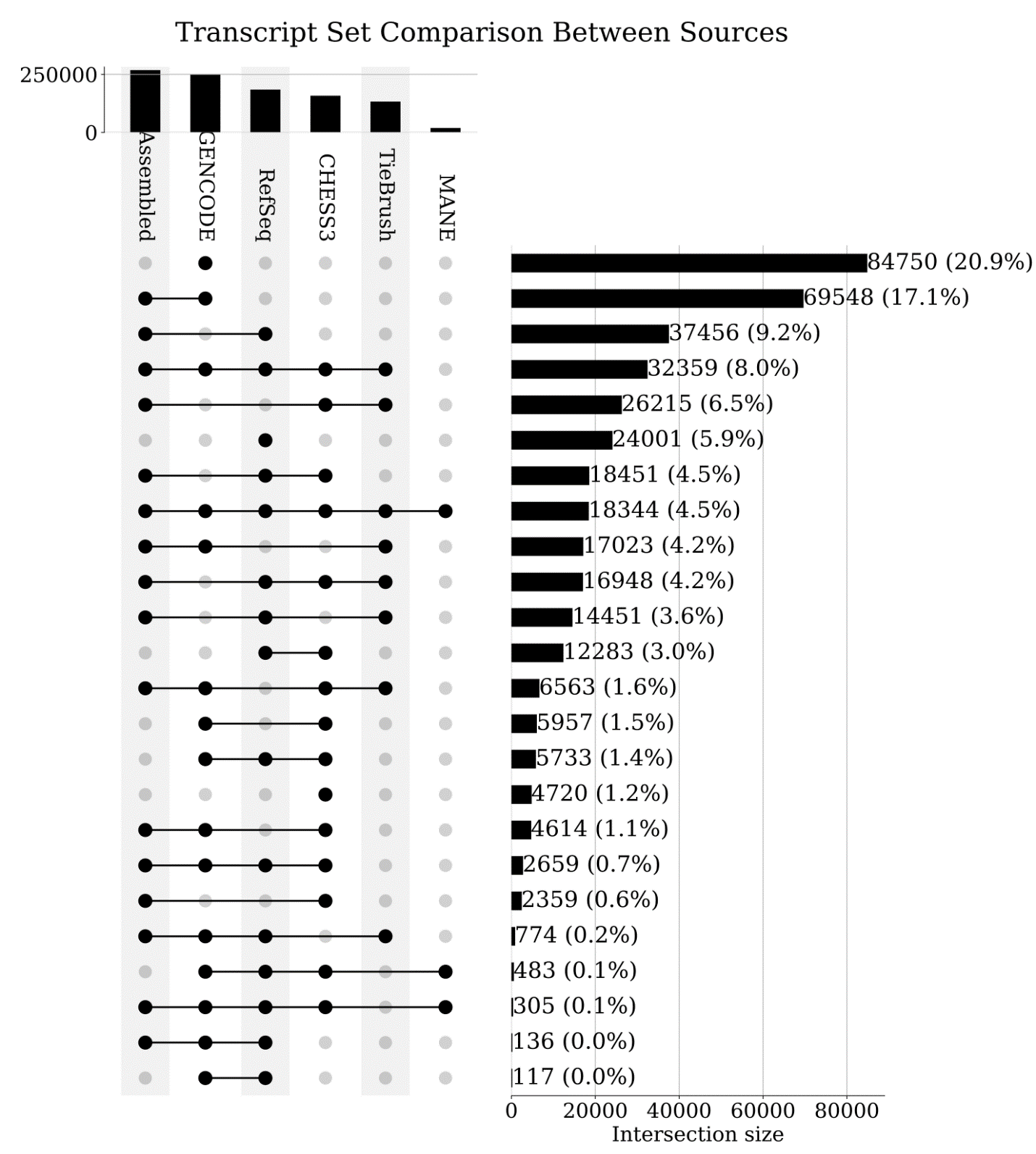
**Figure S3.** Upset plot showing overlap of transcripts in six different datasets: CHESS, RefSeq, GENCODE, MANE, all transcripts assembled from GTEx ("Assembled"), and TieBrush-filtered GTEx isoforms. The plot illustrates the number of transcripts unique to each dataset, as well as the number shared between different datasets. Combinations with no transcripts are excluded from the figure as well as transcripts that were unique to raw assemblies of GTEx and TieBrush. The 136 transcripts that were assembled and present in both RefSeq and GENCODE, but not in CHESS, were removed because they either lacked a valid ORF or because the translated protein was too short compared to other isoforms, as explained in the main text.
