# Supplementary material for: CHESS 3: an improved, comprehensive catalog of human genes and transcripts based on large-scale expression data, phylogenetic analysis, and protein structure
Source: Genome Biol. 2023 Oct 30;24:249. doi: 10.1186/s13059-023-03088-4 (PMC10614308; doi:10.1186/s13059-023-03088-4)
Supplement: Supplementary file 4 — Additional file 4. Review history. [file 13059_2023_3088_MOESM4_ESM.docx]

Review History

**First round of review**

**Reviewer 1**

**Were you able to assess all statistics in the manuscript, including the appropriateness of statistical tests used?**

There are no statistics in the manuscript.

**Comments to author:**

I think this work is important as it provides a new version of CHESS, a high-quality gene model annotation data set generated from a complex pipeline involving RNA-Seq alignments.

This new CHESS release is now available for the telomere-to-telomere CHM13 human genome assembly, along with the older but more commonly used assembly, hg38. Accurate gene model annotations are important for many fields, and are especially important to researchers looking into how alternative RNA splicing patterns are regulated (or not) in humans.

In addition to providing the annotations, the authors also used state-of-the-art protein structure prediction software to evaluate each protein in the annotation set. To explain and illustrate the significance and value of these protein structure predictions, they provide examples comparing expression levels for variants with very different structures.

The article is clearly written, with logical and easy-to-follow organization. My higher-level questions about the new annotation data set were answered, such as, how this new work compares to other annotation sets, how the authors generated the CHESS gene models, and how they view the significances of their work.

These protein structure predictions, combined with a method for scoring the level of RNA-Seq support for alternative introns across the input RNA-Seq data, will make it easier to answer long-standing questions about alternative splicing patterns.

To check that the gene model annotations are usable for this and other purposes, I downloaded some of the annotation files and checked that I could import and visualize them into a genome browser. For this, I used Integrated Genome Browser, a tool that uses the same formats as other popular genome browsers, such as IGV and JBrowse. So, if I could get the data into IGB, probably it would be not too difficult to do the same for the other popular genome browser tools.

There were several GFF files available from the Web site. I decided to use chess3.0.gff.gz, from the top of the list, which references genome assembly hg38.

I opened hg38 in IGB, downloaded the file, and opened it in IGB. After about a minute, I was able to start loading data and looking at the annotations, comparing them the reference annotations that IGB loads by default for this genome assembly version.

Most everything I saw made sense to me. I zoomed in to look at a few favorite examples, including genes mentioned in the article.

However, the GFF size and slowness of loading prompted me to make a new version of the file that uses a more compact browser format - BED14, also called "BED-detail" - combined with bgzip/tabix indexing to make the file random access and smaller/faster to load.

It did not take long to convert the format and check it in IGB.

Based on these experiences, I feel confident that others who want to look at the annotations in their favorite genome browser will also find it easy.

However, I did have two questions about the file:

1. The "extra feature" fields in the GFF file contained interesting information, such as: "num_samples=903;max_tpm=9.496638;assembly_id=ALL_00000008". I wondered: what is "num_samples" referring to? I can guess, but maybe it would be better for the authors to explain this on the Web site, somehow. I also was curious about what the "assembly_id" attribute referred to. Maybe that information is already on the CHESS site, or in the article itself, and I overlooked it?

More thorough documentation about the GFF file would be nice to have.

2. I also wanted to know more about what the values in the gff field 2 represented. For example, some lines contain the string "HAVANA" and others contain the string "havana." I was curious about whether they meant the same thing, or not. For your reference, the values found in the second field from chess3.0.gff.gz included:

BestRefSeq

Curated Genomic

ENSEMBL

FANTOM

Gnomon

HAVANA

RefSeq

StringTie

cmsearch

ensembl

ensembl_havana

havana

tRNAscan-SE

**Reviewer 2**

**Were you able to assess all statistics in the manuscript, including the appropriateness of statistical tests used?**

There are no statistics in the manuscript.

**Comments to author:**

This manuscript, "CHESS 3: an improved, comprehensive catalog of human genes and transcripts based on large-scale expression data, phylogenetic analysis, and protein structure" describes; improvements made to the CHESS database over previous versions, analysis of the CHESS annotation of protein-coding and long non-coding RNA genes and transcripts compared to other widely used gene annotation databases (RefSeq and GENCODE), the use of AlphaFold structural information to identify high quality protein isoforms is the CHESS annotation, including improvements to isoforms current included in the MANE annotation set. The use of CHESS annotation as a reference for the annotation (with Liftoff) of the human T2T-CHM13 genome and chimpanzee genome are also discussed briefly.

The authors describe the use of huge volumes of high quality RNA-seq data from the GTEx project to build transcript models which are then extensively filtered for quality and expression level to produce the final robust set of protein-coding genes and transcripts. Where genes cannot be annotated using the GTEx RNA-seq data, the annotation is supplemented by annotation from RefSeq and GENCODE. The updated methods produce genuine improvements over previous versions of the annotation and the addition of information from AlphaFold also leads to improved discrimination of protein-coding transcripts.

The generation of the CHESS 3 annotation clearly represents an achievement, however, the analysis of the genes and transcripts created for CHESS 3 presented in this manuscript do not seem to maximize it's utility for comparison to the annotation made by RefSeq and GENCODE. In particular, the analysis of the differences between CHESS 3, RefSeq and GENCODE seems a little uneven in its depth.

It would be useful for the authors to expand on the optimization of the expression cutoff selected (minimum number of samples and average TPM threshold of 10 and 1, >=10% of the highest expressed transcript of the gene) given in the supplementary methods section. It would be very useful to see the numbers of transcripts filtered/included at various expression thresholds and the supporting analysis of why these particular thresholds were selected.

Furthermore, it would also be useful to see the kinds of transcript that are included in CHESS as well as those that are filtered. For example, the authors report that "99,202 transcripts...the number of distinct protein sequences produced from these transcripts is 73,767" from which one can infer that alternative splicing both within the CDS and the UTR sequence is being captured within CHESS. It would be useful to understand what kinds of features are being captured; exons skipping vs inclusion, novel promoters, etc. Related to the point above it would also be useful to know how the expression cutoff affects these various classes of possible alternative splicing events to see whether exclusion is relatively consistent or whether particular classes are filtered at different cutoffs eg are exon skips enriched in those transcripts excluded at the lowest thresholds. This would seem to allow the authors to comment on the use of transcriptomic data to test argument for the limited functionality of alternatively spliced transcripts, eg what types of alternative splicing are the noisiest?

Similar analysis would also be extremely useful in identifying what alternative splicing CHESS is capturing that the RefSeq and GENCODE are missing and what particularly noisy splicing they are including.

While the authors describe using RefSeq and GENCODE annotation to ensure coverage of all protein-coding genes it doesn't appear to be the same for the representation of transcripts that contain exons or splice sites that aren't found in the GTEx data due to the incomplete coverage of tissues/cell types/developmental stages. Have the authors investigated to see whether no or low representation of a splice site in the GTEx data explains the transcripts that are present in the RefSeq and GENCODE catalogues but not CHESS or are they transcripts identified by CHESS but rejected during the transcript filtering stage? i.e. does CHESS find the same transcripts as RefSeq and GENCODE but choose to reject them?

Major comments:

Is it valid to include RefSeq NM and XM annotations in the same set for the analysis presented in Table 1 without distinction? CHESS and GENCODE more uniformly annotated sets without this clear separation of annotation method for protein-coding genes. RefSeq are clear that XM and NM are very different things (https://www.ncbi.nlm.nih.gov/books/NBK50679/#RefSeqFAQ.what_is_the_difference_between) and this is reported in the manuscript later (in the section "Comparisons to RefSeq and GENCODE") but this should be reported earlier as it is relevant to other analysis. The authors should also consider whether they should be represented as two distinct sets. The XMs are not curated at all I don't think that there are any RefSeq XM transcripts in the MANE annotation. Does this affect the overlap between the resources eg are RefSeq NMs more likely to agree with CHESS and GENCODE than XM transcripts in Figure 1? Does inlcusion of XMs affect the protein coding gene agreement between GENCODE and RefSeq? Although the MANE set does not cover 100% of protein-coding genes it is reported to be quite close and so I would expect the number of protein-coding genes on the GRCh38 reference to be similarly close.

CHESS is clearly identifying cases where RefSeq and GENCODE should update their decision over the MANE transcript. However, picking a random example (NGRN) from the supplementary table S1 and comparing the CHESS annotation to the RefSeq and GENCODE annotation on the UCSC genome browser it appeared that CHESS had two transcripts at the locus that were the same as those annotated by RefSeq and GENCODE. One transcript had the same CDS, but for the second both RefSeq and GENCODE had a non-coding transcript with the same structure as a CHESS transcript with a CDS. The CHESS-specific CDS used an internal ATG compared to the CDS in all three resources. It looked like RefSeq and GENCODE had annotated the transcript and chosen not to add the CDS, is there any additional evidence that their decision was incorrect?. How many other of the Table S1 examples look similar to this (and might have their validity debated by RefSeq and GENCODE)? It would be useful to calculate where the differences lie between the S1 examples and the RefSeq and GENCODE isoforms to investigate the sources of novelty eg exon skipping or inclusion, alternative ATG, etc.

For the annotation of the T2T-CHM13 and chimpanzee genomes, the authors report simple statistics and for CHM13 point to supplementary tables. This section seems very brief and even slightly out of place, is it possible to bring any more depth to the analysis to the main text?

Minor comments/questions:

Background

"A growing body of evidence suggests that many alternative splicing events do not produce functional proteins [10]" This is a completely reasonable assertion, however, I think this paper was part of a back and forth discussion at the time (eg Blencowe, B.J. (2017) The relationship between alternative splicing and proteomic complexity. Trends in biochemical sciences, 42(6), 407-408.) and seems fair to include the viewpoint arguing broader functionality for alternative splicing.

Results

"Note that GRCh38 also has several hundred alternative scaffolds containing thousands of annotated genes, the vast majority of which are duplicates." While the general point stands, this seems a slightly dismissive way to describe the genes represented on GRC patches that correct sequence errors on the reference genome or represent alternative haplotypes.

The authors report that "that the CHESS database (v2.2) had 224 novel proteins that were missing entirely from both RefSeq and GENCODE." While 5 were now found in RefSeq, GENCODE and MANE are the other transcripts still part of the CHESS 3 dataset?

Have the authors used the methods developed for CHESS to create de novo annotation on other genomes which have less developed reference annotation from other sources?

**Authors Response**

**Point-by-point responses to the reviewers’ comments:**

Reviewer #1:

I think this work is important as it provides a new version of CHESS, a high-quality gene model annotation data set generated from a complex pipeline involving RNA-Seq alignments.

This new CHESS release is now available for the telomere-to-telomere CHM13 human genome assembly, along with the older but more commonly used assembly, hg38. Accurate gene model annotations are important for many fields, and are especially important to researchers looking into how alternative RNA splicing patterns are regulated (or not) in humans.

In addition to providing the annotations, the authors also used state-of-the-art protein structure prediction software to evaluate each protein in the annotation set. To explain and illustrate the significance and value of these protein structure predictions, they provide examples comparing expression levels for variants with very different structures.

The article is clearly written, with logical and easy-to-follow organization. My higher-level questions about the new annotation data set were answered, such as, how this new work compares to other annotation sets, how the authors generated the CHESS gene models, and how they view the significances of their work.

These protein structure predictions, combined with a method for scoring the level of RNA-Seq support for alternative introns across the input RNA-Seq data, will make it easier to answer long-standing questions about alternative splicing patterns.

*Response: thank you for these positive comments about our paper!*

To check that the gene model annotations are usable for this and other purposes, I downloaded some of the annotation files and checked that I could import and visualize them into a genome browser. For this, I used Integrated Genome Browser, a tool that uses the same formats as other popular genome browsers, such as IGV and JBrowse. So, if I could get the data into IGB, probably it would be not too difficult to do the same for the other popular genome browser tools.

There were several GFF files available from the Web site. I decided to use chess3.0.gff.gz, from the top of the list, which references genome assembly hg38.

I opened hg38 in IGB, downloaded the file, and opened it in IGB. After about a minute, I was able to start loading data and looking at the annotations, comparing them the reference annotations that IGB loads by default for this genome assembly version.

Most everything I saw made sense to me. I zoomed in to look at a few favorite examples, including genes mentioned in the article.

However, the GFF size and slowness of loading prompted me to make a new version of the file that uses a more compact browser format - BED14, also called "BED-detail" - combined with bgzip/tabix indexing to make the file random access and smaller/faster to load.

It did not take long to convert the format and check it in IGB.

Based on these experiences, I feel confident that others who want to look at the annotations in their favorite genome browser will also find it easy.

*Response: We thank the reviewer for testing our dataset and for drawing our attention to the missing support for other commonly used genome browsers. We have updated the CHESS dataset to release 3.01, in which we are now providing BED-14 formatted files alongside the GTF/GFF3 files. All files are generated from the same input (chess3.01.gtf) and describe the same contents as one another.*

However, I did have two questions about the file:

1. The "extra feature" fields in the GFF file contained interesting information, such as: "num_samples=903;max_tpm=9.496638;assembly_id=ALL_00000008". I wondered: what is "num_samples" referring to? I can guess, but maybe it would be better for the authors to explain this on the Web site, somehow. I also was curious about what the "assembly_id" attribute referred to. Maybe that information is already on the CHESS site, or in the article itself, and I overlooked it?

More thorough documentation about the GFF file would be nice to have.

*Response: We agree that comprehensive documentation is essential to understanding the contents of the GFF file. As such, we have updated our documentation to provide detailed explanations of all attributes used in the files, including their possible values. The document is available at http://ccb.jhu.edu/chess/index.shtml?t=readme, and the website is already cited in the manuscript. We have also made several improvements to the attributes, such as refining their naming, rounding values, and adding some new ones.*

2. I also wanted to know more about what the values in the gff field 2 represented. For example, some lines contain the string "HAVANA" and others contain the string "havana." I was curious about whether they meant the same thing, or not. For your reference, the values found in the second field from chess3.0.gff.gz included:

BestRefSeq

Curated Genomic

ENSEMBL

FANTOM

Gnomon

HAVANA

RefSeq

StringTie

cmsearch

ensembl

ensembl_havana

havana

tRNAscan-SE

R*esponse: we cleaned up these source labels in our GFF and GTF files to provide more clarity and consistency. The original source labels were all taken directly from RefSeq and GENCODE, which explains their inconsistency; e.g., GENCODE includes both "HAVANA" and "ensembl_havana" which mean the same thing. We now use just four main source labels to represent transcripts:*

*CHESS: This represents all transcripts that are not present in other datasets.*

*MANE: This represents transcripts for which there is an unequivocal agreement between all datasets, and which are in the MANE database.*

*RefSeq: This represents transcripts that match those in RefSeq.*

*GENCODE: This represents all transcripts that match GENCODE and that aren't in either MANE or RefSeq.*

*We hope this helps clarify the source information in the GFF files and improves the usability of our data.*

Reviewer #2:

This manuscript, "CHESS 3: an improved, comprehensive catalog of human genes and transcripts based on large-scale expression data, phylogenetic analysis, and protein structure" describes [...].

It would be useful for the authors to expand on the optimization of the expression cutoff selected (minimum number of samples and average TPM threshold of 10 and 1, >=10% of the highest expressed transcript of the gene) given in the supplementary methods section. It would be very useful to see the numbers of transcripts filtered/included at various expression thresholds and the supporting analysis of why these particular thresholds were selected.

Furthermore, it would also be useful to see the kinds of transcript that are included in CHESS as well as those that are filtered. For example, the authors report that "99,202 transcripts...the number of distinct protein sequences produced from these transcripts is 73,767" from which one can infer that alternative splicing both within the CDS and the UTR sequence is being captured within CHESS. It would be useful to understand what kinds of features are being captured; exons skipping vs inclusion, novel promoters, etc. Related to the point above it would also be useful to know how the expression cutoff affects these various classes of possible alternative splicing events to see whether exclusion is relatively consistent or whether particular classes are filtered at different cutoffs eg are exon skips enriched in those transcripts excluded at the lowest thresholds. This would seem to allow the authors to comment on the use of transcriptomic data to test argument for the limited functionality of alternatively spliced transcripts, eg what types of alternative splicing are the noisiest?

Similar analysis would also be extremely useful in identifying what alternative splicing CHESS is capturing that the RefSeq and GENCODE are missing and what particularly noisy splicing they are including.

While the authors describe using RefSeq and GENCODE annotation to ensure coverage of all protein-coding genes it doesn't appear to be the same for the representation of transcripts that contain exons or splice sites that aren't found in the GTEx data due to the incomplete coverage of tissues/cell types/developmental stages. Have the authors investigated to see whether no or low representation of a splice site in the GTEx data explains the transcripts that are present in the RefSeq and GENCODE catalogues but not CHESS or are they transcripts identified by CHESS but rejected during the transcript filtering stage? i.e. does CHESS find the same transcripts as RefSeq and GENCODE but choose to reject them?

*Response: before we address comments listed as major and minor by the reviewer (below), we address here these general comments regarding the various filtering criteria.*

*We initially assembled 26,335,900 transcripts from the GTEx set of nearly 10,000 RNA-seq experiments. Of those, we retained 987,244 after the TieBrush assembly. (These steps and numbers are described in Methods.) Then we proceeded to apply our ML-based method for assessing confidence of introns and selecting transcripts based on the identified introns. This step generated a set of 235,644 transcripts, to which we added all assembled transcripts that matched RefSeq and GENCODE for a total of 458,079 transcripts. We have added more details about this process in the supplemental text as follows:*

“Selecting novel transcripts based on intron classification and adding assembled RefSeq and GENCODE isoforms resulted in a set of 458,079 candidate isoforms. From the known transcripts in this set, we found 1,268 read-through transcripts, 19,619 transcripts with no start or stop codons found by ORFanage, and 4,670 transcripts with no PhyloCSF++ support, all of which we removed. In the set of novel transcripts, we found 62,076 which had the lowest level of support for the intron chains; an additional 10,457 read-throughs; 20,021 with no start or stop codons found by ORFanage; and 10,201 with no PhyloCSF++ support. These too were removed. The combined removal of transcripts in these categories reduced the set of isoforms down to 341,558. These transcripts were filtered further by removing novel assemblies overlapping loci that cannot be reliably determined from RNA-seq data such as pseudogenes or IG genes. The remaining transcripts included 125,870 novel transcripts and 100,429 that were either present only in GENCODE, or only in RefSeq with an XM or XR tag. Out of these transcripts we only kept 24,725 novel transcripts and 18,855 transcripts from GENCODE or RefSeq only if they had an average expression of at least 1TPM, did not overlap annotated repeats, were expressed in at least 10 samples, and were expressed in more than 10% of the maximum number of samples in which any transcript in the same gene locus was expressed.”

*To further explore the similarities and differences between the datasets, we ran a multi-way comparison between CHESS, MANE, RefSeq and GENCODE, and also included in the comparison all assembled isoforms as well as the TieBrush-filtered set of transcripts. This comparison is presented in Supplemental Figure S3. The supplemental figure shows that we did not observe much evidence for many of the GENCODE-only and RefSeq-only transcripts after the aggressive TieBrush filtering step, and further that the majority were not even assembled in any of the GTEx samples by our protocol. However, we retained all transcripts that were curated by RefSeq (transcripts with IDs starting with an NM or NR tag), or if they were assembled and were present in both RefSeq and GENCODE (see Supplementary Methods, “Adding transcripts from known sources”). A very small number of these transcripts (136, Supplementary Fig S3) were filtered out because they lack a valid translation or because the translated protein is too short compared to other proteins at the same locus.*

Major comments:

Is it valid to include RefSeq NM and XM annotations in the same set for the analysis presented in Table 1 without distinction? CHESS and GENCODE more uniformly annotated sets without this clear separation of annotation method for protein-coding genes. RefSeq are clear that XM and NM are very different things (https://www.ncbi.nlm.nih.gov/books/NBK50679/#RefSeqFAQ.what_is_the_difference_between) and this is reported in the manuscript later (in the section "Comparisons to RefSeq and GENCODE") but this should be reported earlier as it is relevant to other analysis. The authors should also consider whether they should be represented as two distinct sets. The XMs are not curated at all I don't think that there are any RefSeq XM transcripts in the MANE annotation. Does this affect the overlap between the resources eg are RefSeq NMs more likely to agree with CHESS and GENCODE than XM transcripts in Figure 1? Does inlcusion of XMs affect the protein coding gene agreement between GENCODE and RefSeq? Although the MANE set does not cover 100% of protein-coding genes it is reported to be quite close and so I would expect the number of protein-coding genes on the GRCh38 reference to be similarly close.

*Response: we have now clarified our treatment of RefSeq XM and NM transcripts earlier in the manuscript. As the referee points out, we followed RefSeq's recommendation to only include high-confidence (curated) NM transcripts in our analysis, and XM transcripts were treated the same as any other transcript. We have now clarified this strategy in the main and supplemental Methods section as follows:*

*“Note that for CHESS 3, we intentionally included all RefSeq transcripts with the NM tag, while the lower-confidence XM transcripts underwent the same filtering criteria as all other isoforms in the catalog (Supplemental Figure S2).”*

*To investigate the effect of including XM transcripts, we compared the number of all RefSeq protein-coding transcripts (with both NM and XM tags) in various datasets, including our set of all assembled transcripts from GTEx, the TieBrush-filtered set, GENCODE, and CHESS 3. We present this comparison in our new Supplemental Figure 2.*

CHESS is clearly identifying cases where RefSeq and GENCODE should update their decision over the MANE transcript. However, picking a random example (NGRN) from the supplementary table S1 and comparing the CHESS annotation to the RefSeq and GENCODE annotation on the UCSC genome browser it appeared that CHESS had two transcripts at the locus that were the same as those annotated by RefSeq and GENCODE. One transcript had the same CDS, but for the second both RefSeq and GENCODE had a non-coding transcript with the same structure as a CHESS transcript with a CDS. The CHESS-specific CDS used an internal ATG compared to the CDS in all three resources. It looked like RefSeq and GENCODE had annotated the transcript and chosen not to add the CDS, is there any additional evidence that their decision was incorrect?. How many other of the Table S1 examples look similar to this (and might have their validity debated by RefSeq and GENCODE)? It would be useful to calculate where the differences lie between the S1 examples and the RefSeq and GENCODE isoforms to investigate the sources of novelty eg exon skipping or inclusion, alternative ATG, etc.

*Response: both RefSeq and GENCODE provide little to no explanation for why some of the transcripts at protein-coding loci are labeled as non-coding. We made a deliberate decision to require all protein-coding transcripts that we kept in CHESS to have a valid CDS in our catalog, as described in the text. Our analysis supported some of the transcripts that GENCODE and/or RefSeq tagged as non-coding within protein-coding genes. Our program, ORFanage, identified these transcripts to have valid ORFs, so we kept them as coding in our set.*

*We have also explored the alternative splicing (AS) events between the datasets, and quantified several types of AS events as compared to MANE. We report these findings in Supplementary Table 5 and have updated the main text as follows:*

*“If we exclude duplicate amino-acid sequences, the number of distinct protein sequences produced from these transcripts is 73,767 (Table 1), capturing over 17,000 alternative translation start and stop sites as well as almost 24,000 exon-skipping and 22,000 intron retention events (Supplementary Table S5).”*

For the annotation of the T2T-CHM13 and chimpanzee genomes, the authors report simple statistics and for CHM13 point to supplementary tables. This section seems very brief and even slightly out of place, is it possible to bring any more depth to the analysis to the main text?

*Response: we agree that the annotation of the complete human genome CHM13 deserves more attention. Our focus in this study was to update and validate the CHESS protocol and database. We believe that it is important to acknowledge the significance of CHM13 as the first complete human reference genome, and our decision to present simple statistics and supplementary tables was to provide users with immediate access to our database on this assembly. In response to this comment, we have begun looking at some of these differences in more detail, but it quickly became apparent that a discussion of the CHM13 annotation would really be about the differences between CHM13 and GRCh38, which are numerous and complex, but which are not really the subject of this manuscript. We hope to expand our analysis of the CHM13 annotation in the future.*

*Regarding the comparison with the chimpanzee genome, we agree that this section is not directly relevant to the main focus of our study, and we have removed it from the manuscript.*

Minor comments/questions:

"A growing body of evidence suggests that many alternative splicing events do not produce functional proteins [10]" This is a completely reasonable assertion, however, I think this paper was part of a back and forth discussion at the time (eg Blencowe, B.J. (2017) The relationship between alternative splicing and proteomic complexity. Trends in biochemical sciences, 42(6), 407-408.) and seems fair to include the viewpoint arguing broader functionality for alternative splicing.

*Response: we now include the above-mentioned citation as follows:*

*“Although opinions differ on the topic [10], a growing body of evidence suggests that many alternative splicing events do not produce functional proteins [11].”*

Results

"Note that GRCh38 also has several hundred alternative scaffolds containing thousands of annotated genes, the vast majority of which are duplicates." While the general point stands, this seems a slightly dismissive way to describe the genes represented on GRC patches that correct sequence errors on the reference genome or represent alternative haplotypes.

*Response: we have updated our discussion to include this point, as follows:*

*“Note that GRCh38 also has several hundred alternative scaffolds containing thousands of annotated genes, the vast majority of which are duplicates, although some represent alternative haplotypes.”*

The authors report that "that the CHESS database (v2.2) had 224 novel proteins that were missing entirely from both RefSeq and GENCODE." While 5 were now found in RefSeq, GENCODE and MANE are the other transcripts still part of the CHESS 3 dataset?

*Response: Indeed, in the main text we showed that 5 genes originally discovered as part of the CHESS project now made it into agreed-upon curated datasets (namely MANE). We have now included more details regarding these 224 genes, as described below in the main manuscript:*

*“We reported previously [5] that the CHESS database (v2.2) had 224 novel proteins that were missing entirely from both RefSeq and GENCODE. We investigated recent releases of both databases (v110 and v41 respectively) and found that many of the previously novel protein-coding genes are now included in other datasets: 53 in GENCODE, 23 in RefSeq, and 5 in MANE (detailed in Table 3). With the new approaches described in the current report, we assembled 185 of the previously reported genes, and most of them were filtered out by our newer, more stringent criteria. The current release of the dataset includes only 47 of the 224 genes that were novel to the CHESS 2 dataset.”*

Have the authors used the methods developed for CHESS to create de novo annotation on other genomes which have less developed reference annotation from other sources?

*Response: We have not yet had time to attempt to build a similar catalog for any other genomes, although our methods could apply to any genome for which sufficient RNA-seq resources are available.*

**Second round of review**

**Reviewer 2**

No additional questions or comments.
